# Cannabidiol as a novel therapeutic agent in breast cancer: evidence from literature

**DOI:** 10.1186/s12885-025-14175-z

**Published:** 2025-04-24

**Authors:** Mojtaba Esmaeli, Maryam Dehghanpour Dehabadi, Ali Asghar Khaleghi

**Affiliations:** 1 Cellular and Molecular Research Center, Gerash University of Medical Sciences, Gerash, Iran; 2 Department of Medical Emergencies, School of Medicine, Fasa University of Medical Science, Fasa, Iran

**Keywords:** Cannabidiol (CBD), Breast cancer, Triple-negative breast cancer (TNBC), Apoptosis, Metastasis, Molecular pathways, Clinical translation

## Abstract

**Background:**

Breast cancer is one of the most prevalent cancers worldwide, posing significant challenges due to its heterogeneity and the emergence of drug resistance. Cannabidiol (CBD), a non-psychoactive compound derived from *Cannabis sativa*, has recently gained attention for its potential therapeutic effects in breast cancer.

**Objective:**

This review aims to evaluate the antitumor effects of CBD in breast cancer treatment by synthesizing preclinical and clinical evidence, elucidating its mechanisms of action, and exploring its translational potential.

**Methods:**

A systematic review was conducted following PRISMA guidelines. A comprehensive search was performed across PubMed, Google Scholar, Web of Science, and Scopus databases, using keywords such as “Cannabidiol,” “CBD,” “Breast Cancer,” “Therapeutic Agent,” and “Antitumor Effects.” A total of 1,191 articles were initially identified. After duplicate removal and eligibility screening, 34 studies published between 1998 and 2025 were selected, including in vitro, in vivo, and clinical investigations. Studies were assessed based on PRISMA recommendations, considering inclusion criteria such as CBD’s impact on apoptosis, cell proliferation, tumor progression, and molecular mechanisms.

**Results:**

CBD demonstrated significant anticancer effects, including induction of apoptosis, inhibition of cell proliferation, suppression of metastasis, and modulation of the tumor microenvironment. Mechanistically, CBD modulates key pathways such as PI3K/Akt, mTOR, and PPARγ and interacts with CB1, CB2, and non-cannabinoid receptors. Preclinical studies showed CBD’s efficacy, particularly in triple-negative breast cancer (TNBC), while limited clinical trials highlighted its potential as an adjunct to conventional therapies.

**Conclusion:**

CBD offers a promising therapeutic approach for breast cancer, especially for aggressive subtypes like TNBC. However, challenges such as variability in study design, lack of standardized protocols, and limited clinical validation hinder its clinical application. Future research should focus on conducting robust clinical trials, identifying predictive biomarkers, and optimizing combinatorial therapies to integrate CBD into personalized cancer treatment strategies.

## Introduction

Breast cancer is one of the most prevalent malignancies among women worldwide, characterized by its heterogeneity and diverse subtypes, each requiring distinct therapeutic strategies [1]. Despite significant advancements in treatment modalities, challenges persist, including resistance to existing therapies and the adverse effects associated with conventional treatments like chemotherapy and radiotherapy [2]. The search for novel therapeutic agents that can complement or enhance existing therapies while minimizing side effects has led to increased interest in plant-derived compounds, particularly cannabinoids [3].

Cannabidiol (CBD), a non-psychoactive phytocannabinoid derived from Cannabis sativa, has garnered attention due to its potential anticancer properties [4, 5]. Recent studies have highlighted CBD’s ability to inhibit tumor growth, induce apoptosis, and suppress metastasis in preclinical models of breast cancer [6]. CBD exerts its effects through multiple mechanisms, including modulation of the endocannabinoid system, induction of apoptosis, inhibition of cell proliferation, and suppression of metastasis [6, 7]. It interacts with cannabinoid receptors (CB1 and CB2) and non-cannabinoid receptors such as TRPV1 and PPARγ, contributing to its anticancer properties [8].

Preclinical studies have demonstrated the efficacy of CBD in reducing tumor growth and metastasis in various breast cancer models, particularly in triple-negative breast cancer (TNBC), which lacks targeted therapies [9]. Clinical studies have begun to explore CBD’s potential as a complementary therapy in breast cancer patients, with promising results in symptom management and quality of life improvement [10].

This literature review aims to synthesize recent findings on the antitumor effects of CBD in breast cancer, elucidating its mechanisms of action and potential clinical applications. By examining the current evidence, we seek to contribute to the development of novel therapeutic strategies that could enhance the effectiveness of existing treatments and improve patient outcomes.

## Materials and methods

### Literature search

A comprehensive literature search was conducted using electronic databases including PubMed, Google Scholar, Web of Science, and Scopus. The search terms used were “Cannabidiol,” “CBD,” “Breast Cancer,” “Therapeutic Agent,” and “Antitumor Effects.” The search was limited to articles published in English from 1998 to 2025. Boolean operators (AND, OR) were used to combine terms. Additional articles were identified through manual searches of reference lists from relevant studies.

### Eligibility criteria

#### Inclusion criteria

Studies were included if they met the following criteria:

Studies that investigated the effects of CBD on breast cancer cells or animal models.

Clinical studies that included breast cancer patients receiving CBD as part of their treatment regimen.

Studies published in peer-reviewed journals in the last 27 years.

Articles must have been published in English.

Provided data on the mechanisms of action, efficacy, and safety of CBD in breast cancer treatment.

#### Exclusion criteria

Studies were excluded if they:

Studies that did not focus on breast cancer.

Studies with insufficient data or incomplete results.

Studies were not available in full text.

### Review process

The review process involved several key steps to ensure the thoroughness and reliability of the findings:

1. **Initial Screening**: Titles and abstracts of all identified studies were screened to determine their eligibility based on the inclusion and exclusion criteria.
2. **Full-Text Review**: Full texts of potentially eligible studies were retrieved and reviewed in detail to confirm their inclusion in the analysis.
3. **Quality Assessment**: The methodological quality of the included studies was assessed using appropriate quality assessment tools (e.g., Cochrane Risk of Bias Tool, Newcastle-Ottawa Scale). Studies were rated as high, moderate, or low quality based on criteria such as randomization, blinding, and completeness of outcome data.
4. **Data Synthesis**: Extracted data were synthesized to provide a comprehensive overview of the effects of CBD on breast cancer. This synthesis included qualitative descriptions of the study findings without performing a meta-analysis.

**Fig. 1:**
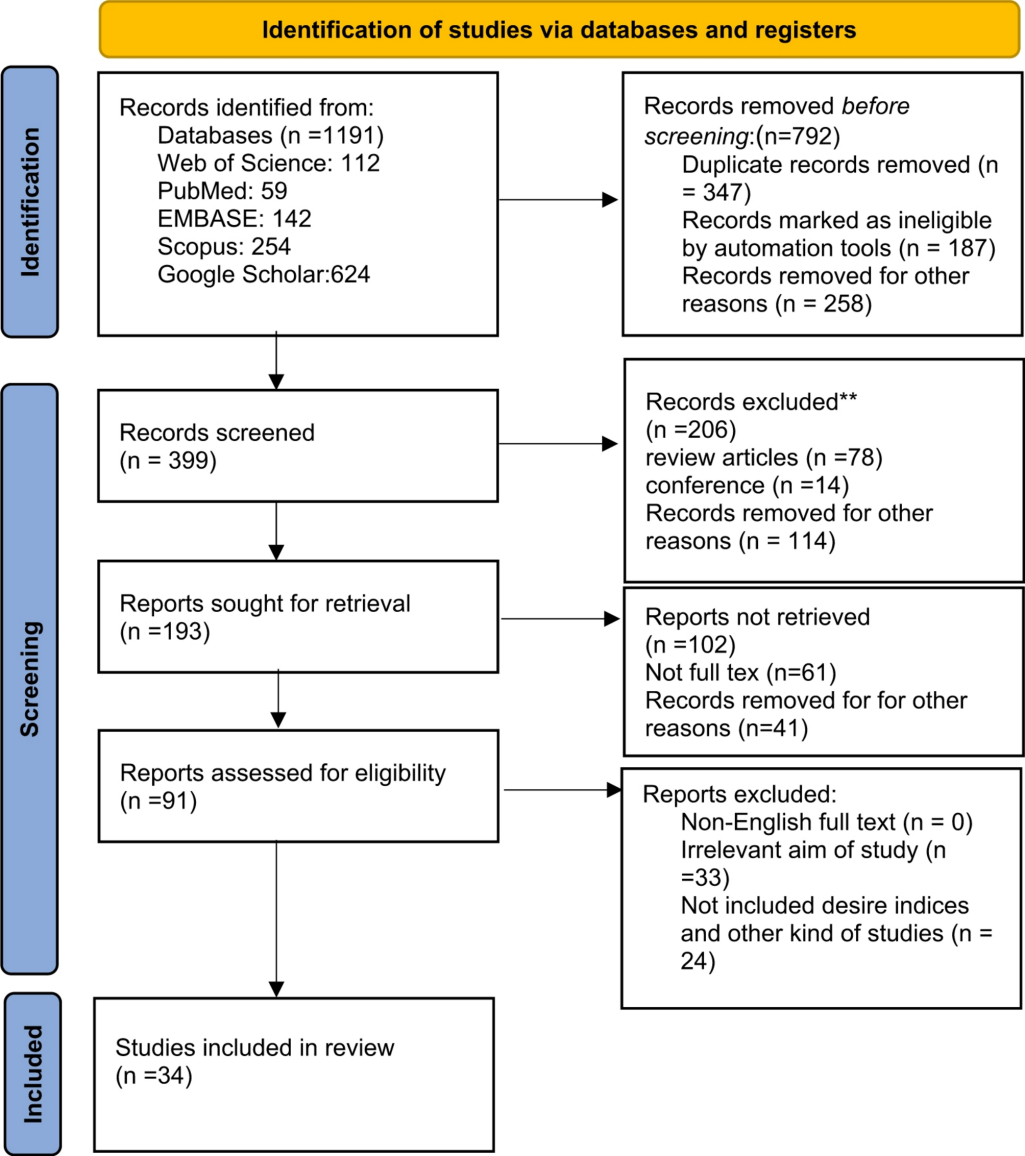
The flow diagram of the selection of sources searched in this systematic review

Figure 1 presents a PRISMA 2020 flow diagram illustrating the search process, including the number of studies identified, screened, assessed for eligibility, and included in the final review.

### Risk of bias and quality assessment

To ensure the reliability of the included studies, a systematic quality assessment was performed using the Cochrane Risk of Bias Tool for randomized trials and the Newcastle-Ottawa Scale for observational studies. The following parameters were considered:

- **Selection Bias**: Assessment of randomization and allocation concealment.
- **Performance Bias**: Evaluation of blinding methodologies used.
- **Detection Bias**: Analysis of outcome assessment blinding.
- **Attrition Bias**: Consideration of incomplete outcome data and follow-up rates.
- **Reporting Bias**: Examination of selective outcome reporting. Each study was rated as having a high, moderate, or low risk of bias based on these criteria.

### Data extraction and review process

#### Data extraction

Data were meticulously extracted from the selected studies using a standardized data extraction form. The following key information was obtained from each study:

- **Study Design**: The type of study conducted (e.g., in vitro, in vivo, clinical trials).
- **Sample Size**: The number of participants or samples included in the study.
- **Type of Breast Cancer**: Specific subtypes of breast cancer being investigated.
- **CBD Dosage and Administration**: The dosage, frequency, and method of cannabidiol (CBD) administration.
- **Outcomes Measured**: The primary and secondary outcomes assessed in the study, such as tumor size reduction, apoptosis induction, cell proliferation inhibition, and metastasis prevention.
- **Key Findings**: Main results and conclusions drawn from the study.

Two independent reviewers conducted the data extraction process to ensure accuracy and consistency. Any discrepancies between the reviewers were resolved through discussion and consensus, or by consulting a third reviewer if necessary. The selected studies are summarized in Tables 1, 2 and 3.

**Table 1:** CBD as monotherapy in breast Cancer treatment

	Publication Year	Study Type	Cell Line	Breast Cancer Type (Subtype & Biomolecular Markers)	CBD Dosage and Administration	Outcomes Measured	Key Findings	Molecular Pathways	References
The endogenous cannabinoid anandamide inhibits human breast cancer cell proliferation	1998	In vitro (cell lines)	MCF-7, EFM-19	Luminal A (ER+, PR+, HER2-)	Anandamide (AEA): 0.5–10 µM (in vitro) for MCF-7 and EFM-19 cells. IC50 values between 0.5 and 1.5 µM. Maximal inhibition at 5–10 µM.	Cell proliferation inhibition, DNA synthesis inhibition, reduction in S phase cells	Anandamide potently and selectively inhibits human breast cancer cell proliferation through CB1-like receptor-mediated inhibition of endogenous prolactin action at the level of the prolactin receptor	CB1-mediated inhibition of prolactin signaling	[11]
Cannabidiol as a novel inhibitor of Id-1 gene expression in aggressive breast cancer cells	2007	In vitro (cell lines), Reporter Assay	MDA-MB-231, MDA-MB-436, MDA-MB231-Id-1	Triple-Negative Breast Cancer (TNBC) (ER-, PR-, HER2-)	CBD: 0.1, 1.0, or 1.5 µM (in vitro)	Cell proliferation, cell invasion, Id-1 protein expression (Western blot), Id-1 mRNA expression (PCR), Id-1 promoter activity (reporter assay)	CBD inhibited cell proliferation and invasion in MDA-MB-231 cells. CBD downregulated Id-1 mRNA and protein expression in a concentration-dependent manner by inhibiting the Id-1 gene at the promoter level. CBD did not inhibit invasiveness in cells ectopically expressing Id-1.	Id-1 suppression, apoptotic pathways	[8]
Role of Cannabinoid and Vanilloid Receptors in Invasion of Human Breast Carcinoma Cells	2012	Preclinical (in vitro)	MDA-MB-231	Triple-Negative Breast Cancer (TNBC) (ER-, PR-, HER2-)	MA (2, 10, 20 µM), ACEA (50, 100, 200 nM), OLDA (50, 100, 200 nM), CB65 (50, 100, 200 nM), AM251 (10, 20, 40 nM), Capsazepine (5, 10, 15 µM). Selection based on reported Ki/EC50.	Cell proliferation (MTT assay), invasion (Matrigel assay), VEGF levels (ELISA), protein expression (Western blot) (COX-2, MMP-2, CB1, CB2, TRPV1)	Activation of CB1 and vanilloid receptors reduces invasion of MDA-MB-231 cells. CB1 activation decreases MMP2 expression. CB2 activation increases cell invasion and MMP2 expression. Reduced VEGF levels observed with MA, ACEA, CB65, and AM251. Elevated COX-2 expression observed in cells treated with agonists. Blocking CB1 reversed MA inhibition of invasion, while blocking CB2 further reduced invasion. Blocking TRPV1 partially reversed MA inhibition of invasion, however capsazepine alone increased invasion.	CB1 reduces invasion via MMP2 inhibition; CB2 increases invasion; TRPV1 modulates COX-2	[12]
Cannabidiol enhances xenobiotic permeability through the human placental barrier by direct inhibition of breast cancer resistance protein: an ex vivo study	2013	In vitro (cell lines), Ex vivo (human placental perfusion)	BeWo, Jar, MCF7/P-gp cells	Hormone Receptor-Positive Breast Cancer (MCF-7)	CBD: 10–25 µM (in vitro), 15 µM (ex vivo) Glyburide (200 ng/mL) was introduced to maternal and fetal compartments through a recirculating 2 h	BCRP perfusion, dependent mitoxantrone efflux, glyburide transport, female/male ratio of glyburide concentrations	CBD inhibited BCRP-dependent mitoxantrone efflux in a concentration-dependent manner. CBD increased the female/male ratio of glyburide concentrations (1.3 ± 0.1 vs. 0.8 ± 0.1 at 120 min of perfusion, *P* <.001).	Direct inhibition of breast cancer resistance protein (BCRP)	[13]
The use of styrene maleic acid nanomicelles encapsulating the synthetic cannabinoid analog WIN55, 212-2 for the treatment of cancer	2015	In vitro (cell lines)	MDA-MB-231, MCF-7, and PC3	Triple-Negative Breast Cancer (TNBC) (ER-, PR-, HER2-), Hormone Receptor-Positive Breast Cancer (MCF-7),	SMA-WIN Micelles & Free WIN-55,212-2: 0–10 µM concentrations for 72 h.	Cell growth inhibition, IC50 values, Micelle characterization (size, charge, drug loading, drug release rate)	SMA-WIN micelles synthesized with ~ 15% loading, ~ 132.7 nm diameter. Both free and micellar WIN inhibited cell growth in all cell lines, with equal cytotoxicity (IC50 values in the µM range). Slower drug release rate at physiological pH	Micelle-mediated drug delivery improving CBD bioavailability	[14]
Cannabidiol (CBD) is a novel inhibitor for exosome and microvesicle (EMV) release in cancer	2018	In vitro	Prostate cancer (PC3), Hepatocellular carcinoma (HEPG2), Breast adenocarcinoma (MDA-MB-231)	Triple-Negative Breast Cancer (TNBC) (ER-, PR-, HER2-) (MDA-MB-231)	CBD at concentrations of 1 µM and 5 µM, administered directly to cell cultures	EMV release, mitochondrial function, STAT3 and prohibitin expression	CBD significantly reduced exosome and microvesicle release in all studied cancer cell lines in a dose-dependent manner. It modulated mitochondrial function and sensitized cancer cells to chemotherapy, suggesting its potential as a therapeutic agent	STAT3 & prohibitin modulation	[15]
Novel mechanism of cannabidiol-induced apoptosis in breast cancer cell lines	2018	Preclinical (in vitro)	T-47D, MDA-MB-231	ER-positive (T-47D), Triple-Negative Breast Cancer (TNBC) (ER-, PR-, HER2-) (MDA-MB-231)	1–7 mM CBD for T-47D, 1–5 mM CBD for MDA-MB-231 (in serum-free media for viability assays, other concentrations for different assays as described in methods)	Cell viability (MTT assay), morphological changes, DNA fragmentation, ELISA apoptosis assay, protein expression (mTOR, Cyclin D1, PPARγ), Immunoprecipitation, Immunocytochemistry	CBD inhibits cell survival and induces apoptosis in both ER-positive (T-47D) and triple-negative (MDA-MB-231) breast cancer cell lines in a dose-dependent manner. CBD down-regulates mTOR and Cyclin D1 and up-regulates and localizes PPARγ protein expression in the nuclei and cytoplasm. Interplay among PPARγ, mTOR, and Cyclin D1 favors apoptosis induction in both subtypes.	PPARγ activation, mTOR inhibition, Cyclin D1 downregulation	[16]
UPLC-MS Analysis of Cannabis sativa Using Tetrahydrocannabinol (THC), Cannabidiol (CBD), and Tetrahydrocannabinolic Acid (THCA) as Marker Compounds: Inhibition of Breast Cancer Cell Survival and Progression	2019	In vitro (cell lines), CAM assay, UPLC-MS analysis	MDA-MB-231	Triple-Negative Breast Cancer (TNBC) (ER-, PR-, HER2-) (MDA-MB-231)	C. sativa DCM extract: 10–100 µg/mL for 24/48 hrs (MTT assay); 40 µg in CAM assay	Cell viability, IC50, Angiogenic index (CAM assay), UPLC-MS analysis (CBD, THC, THCA content)	C. sativa DCM extract: Inhibited MDA-MB-231 cell growth (IC50 = 27.8 ± 5.0 µg/mL after 48 h). Inhibited VEGF-induced angiogenesis in CAM assay. UPLC-MS confirmed presence of CBD, THC, and THCA.	VEGF inhibition, apoptotic pathways	[17]
Molecular targets of minor cannabinoids in breast cancer: in Silico and in vitro studies	2024	In silico and in vitro	MCF-7	ER + breast cancer (MCF-7)	Not specified for in vitro testing	Aromatase activity, ERα/ERβ modulation, AR interaction, inverse agonist/antagonist properties	Minor cannabinoids showed potential as weak aromatase inhibitors, ERα antagonists with inverse agonist properties, and AR antagonists. Some acted as ER agonists. These findings suggest therapeutic potential for minor cannabinoids in modulating key targets in ER + breast cancer.	ERα inhibition, AR modulation, inverse agonist effects	[18]
CBD inhibits in vivo development of human breast cancer tumors	2023	In vitro, In vivo	MCF-7	ERα+ (MCF-7)	CBD at varying concentrations; administered to cell cultures and mice	Tumor growth inhibition, apoptosis induction, metastasis reduction	CBD significantly inhibited the growth and progression of human breast cancer tumors in both cell cultures and animal models. The treatment led to apoptosis induction, reduced proliferation, and decreased metastasis, suggesting CBD’s potential as a therapeutic agent.	Apoptotic pathways, tumor microenvironment modulation	[19]
Investigation of the effects of the endogenous cannabinoid anandamide on luminal a breast cancer cell line MCF-7	2022	In vitro (cell line)	MCF-7 (Luminal A Breast Cancer)	Luminal A Breast Cancer (ER+, PR+, HER2-)	Anandamide (AEA): 25 µM, 50µM and 75 µM concentrations for 24 h (MTT assay); Optimum concentration 25 μm used to analyse cell	kinetic parameters at Cell viability (MTT & xCELLigence), Mitotic Index, Labelling Index, Apoptotic Index	Anandamide (AEA): Optimum concentration 25 µM decreased cell viability in time-dependent manner, decreased cells in mitotic and synthesis phase, increased apoptotic cells.	CB1 activation, apoptosis induction	[20]
Cannabidiol modulates mitochondrial redox and dynamics in MCF7 cancer cells: A study using fluorescence lifetime imaging microscopy of NAD (P) H	2021	In vitro	MCF7	Luminal A Breast Cancer (ER+, PR+, HER2-)	CBD at concentrations of 1, 5, and 10 µM, administered directly to cell cultures	Mitochondrial function, redox status, cell viability, apoptosis	CBD alters mitochondrial redox state and dynamics, leading to reduced cell viability and induced apoptosis in MCF7 cells. The study used FLIM of NAD(P)H to reveal metabolic changes caused by CBD.	Oxidative stress, mitochondrial dysfunction	[21]
Cannabidiol for Scan-Related Anxiety in Women With Advanced Breast Cancer	2024	Randomized Clinical Trial	-	Advanced Breast Cancer	CBD dosage not specified (patients received either CBD or placebo)	Anxiety levels related to imaging scans	CBD did not significantly reduce scan-related anxiety but lowered general anxiety levels without adverse effects.	Neurotransmitter modulation, anxiolytic effects	[22]

**Table 2:** CBD in combination therapy for breast cancer

	Publication Year	Study Type	Cell Line	Breast Cancer Type (Subtype & Biomolecular Markers)	CBD Dosage and Administration	Outcomes Measured	Key Findings	Molecular Pathways	References
Synthetic cannabinoid receptor agonists inhibit tumor growth and metastasis of breast cancer	2009	Preclinical (in vitro and in vivo)	In vitro: MDA-MB231, MDA-MB231-luc, MDA-MB468; In vivo: SCID mice, PyMT transgenic mice	Triple-Negative Breast Cancer (TNBC) (ER-, PR-, HER2-)	JWH-133 (CB2 agonist) and WIN-55,212-2 (CB1/CB2 agonist): 5 mg/kg/day i.p. in mice. In vitro concentrations not specified	Cell proliferation, migration, tumor growth, lung metastasis, apoptosis, COX-2/PGE2 signaling, c-Fos/c-Jun/Cdc42 activity, angiogenesis, Ki67 expression, CD31 expression	CB1 and CB2 receptors are overexpressed in primary human breast tumors. Synthetic agonists JWH-133 and WIN-55,212-2 inhibit cell proliferation and migration in vitro, reduce tumor growth and lung metastasis in vivo (40–50% reduction in tumor growth, 65–80% reduction in lung metastasis), delay mammary gland tumors in PyMT mice. Effects reversed by CB1/CB2 antagonists. They mediate tumor-suppressive effects via COX-2/PGE2 signaling and induction of apoptosis. They also modulate downstream molecules c-Fos, c-Jun, and Cdc42.	COX-2/PGE2 suppression, apoptotic pathways, c-Fos/c-Jun modulation	[23]
Cannabinoids reduce ErbB2-driven breast cancer progression through Akt inhibition	2010	In vivo (MMTV-neu mice), Ex vivo (human tumor samples)	MMTV-neu mice; Human tumors (ErbB2-positive)	HER2-Positive Breast Cancer (ErbB2-positive)	THC: Peritumoral administration (Dosage not specified). JWH-133: Peritumoral administration (Dosage not specified).	Tumor growth, tumor number, lung metastases (incidence & severity), cell proliferation (Ki67), apoptosis (cleaved caspase 3), angiogenesis (CD31), MMP activity, Akt signaling	THC and JWH-133: Reduced tumor growth, tumor number, and lung metastases. Decreased cell proliferation, induced apoptosis, and impaired angiogenesis in tumors. Downregulated Akt signaling pathway. 91% of ErbB2-positive human breast tumors express CB2 receptors.	CB2 activation, Akt pathway inhibition, apoptosis induction	[24]
Bone cell-autonomous contribution of type 2 cannabinoid receptor to breast cancer-induced osteolysis	2015	Preclinical (in vitro and in vivo)	Human MDA-MB-231, MCF7, Mouse 4T1 breast cancer cells, Calvarial bones of 2-day-old mice, Bone marrow macrophages	Triple-Negative Breast Cancer (TNBC) (ER-, PR-, HER2-) (MDA-MB-231), Hormone Receptor-Positive Breast Cancer (MCF7), Triple-Negative Breast Cancer (4T1)	CB2 agonists (e.g., HU308, JWH-133) administered in vitro and in vivo	Cell viability, osteoclastogenesis, osteolysis, osteoblast growth/differentiation, PI3K/AKT activity, Caspase-3 activity.	High micromolar concentrations of HU308 and JWH133 reduce the viability of breast cancer cells. Low nanomolar concentrations of HU308 and JWH133 enhance breast cancer cell-induced osteoclastogenesis and exacerbate osteolysis. HU308 and JWH133 induce PI3K/AKT activity in a CB2-dependent manner. CB2-selective activation and antagonism have potential efficacy in cancer-associated bone disease, but further studies are warranted. In the presence of conditioned medium from breast cancer cells, HU308 and JWH133 enhanced parathyroid hormone-induced osteoblast differentiation and the ability to support osteoclast formation. CB2 Activation enhances Breast Cancer-Induced Bone cell Activity and osteolysis via the PI3K/AKT pathway.	CB2 activation via PI3K/AKT pathway, bone metastasis modulation	[25]
Modulation of breast cancer cell viability by a cannabinoid receptor 2 agonist, JWH-015, is calcium dependent	2016	Preclinical (in vitro and in vivo)	4T1, MCF7	Triple-Negative Breast Cancer (TNBC) (ER-, PR-, HER2-) (4T1), Hormone Receptor-Positive Breast Cancer (MCF7)	JWH-015, dose-dependent (e.g., A50 values: ~2.8 µM for 4T1, ~ 4.16 µM for MCF7)	Viability, apoptosis, metastasis, calcium flux, MAPK/ERK signaling	JWH-015 significantly reduced tumor viability and metastasis in vivo and induced apoptosis in vitro via a calcium-dependent mechanism. Effects were independent of CB1, GPR55, TRPV1/TRPA1 receptors, and Gαi signaling. MAPK/ERK modulation was implicated.	MAPK/ERK modulation, calcium-dependent apoptosis	[26]
Novel role of cannabinoid receptor 2 in inhibiting EGF/EGFR and IGF-I/IGF-IR pathways in breast cancer	2016	Preclinical (in vitro and in vivo)	SUM159, MDA-MB231, MCF-7	ERα- (SUM159, MDA-MB-231), ERα+ (MCF-7)	JWH-015 (CNR2 specific agonist): In vitro - concentrations not specified; In vivo − 10 mg/Kg peri-tumoral injection for 4 weeks	Cell migration, cell invasion, NF-kB activation, EGFR/IGF-IR activation, tumor volume, tumor weight, STAT3/AKT/ERK activation, MMP secretion	CNR2 activation inhibits EGF/EGFR and IGF-I/IGF-IR signaling, reducing migration and invasion in both ERα- and ERα + breast cancer cells. In vivo, JWH-015 reduces tumor growth and inhibits EGFR/IGF-IR activation. Higher CNR2 expression correlates with better recurrence-free survival in breast cancer patients.	CNR2 activation inhibits EGF/EGFR and IGF-I/IGF-IR signaling	[27]
Appraising the “entourage effect”: Antitumor action of a pure cannabinoid versus a botanical drug preparation in preclinical models of breast cancer	2018	Preclinical (in vitro and in vivo)	MCF-7 and T47D (ER+, PR+, HER2−); BT474 and HCC1954 (HER2+); MDA-MB-231 and SUM 159 (ER−, PR−, HER2−)	ER+/PR+, HER2+, Triple-Negative Breast Cancer (TNBC) (ER-, PR-, HER2-)	Pure THC and Botanical Drug Preparation (BDP) administered orally in mice (45 mg/kg THC)	Cell viability, tumor growth, interaction with standard therapies (e.g., tamoxifen, lapatinib, cisplatin)	BDP was more potent than pure THC in reducing cell viability and tumor growth. The “entourage effect” suggests that compounds in BDP enhance efficacy. No negative interactions with standard therapies were observed.	Entourage effect enhancing cannabinoid efficacy	[28]
Antitumor activity of abnormal cannabidiol and its analog O-1602 in taxol-resistant preclinical models of breast cancer	2019	In vitro, In vivo	MDA-MB-231 and MCF-7	Triple-Negative Breast Cancer (TNBC) (ER-, PR-, HER2-)Triple-negative breast cancer (TNBC), ERα+ (MCF-7)	Abnormal cannabidiol (Abn-CBD) and O-1602 at varying concentrations, administered to cell cultures and animal models, 2 µM (in zebrafish xenograft model)	Cell viability, apoptosis induction, tumor growth inhibition, gene expression analysis	Abn-CBD and O-1602 significantly inhibited proliferation of Taxol-resistant breast cancer cells, induced apoptosis, and reduced tumor growth in xenograft models. They enhanced the efficacy of Taxol chemotherapy, suggesting potential to overcome drug resistance in breast cancer treatment. The compounds acted through non-CB1/CB2 cannabinoid receptors, highlighting a novel mechanism of action.	Non-CB1/CB2 mediated apoptosis and chemotherapy sensitization	[29]
Cannabinoid combination induces cytoplasmic vacuolation in MCF-7 breast cancer cells	2020	Preclinical (in vitro)	MCF-7	Luminal A (ER+, PR+, HER2-)	C6 combination (THC, CBD, CBG, CBN); 40–60 µM total concentration	Cell cycle arrest, apoptosis, cytoplasmic vacuolation, lipid accumulation, lysosomal changes	The C6 combination induced cytoplasmic vacuolation through mechanisms involving autophagy and paraptosis. At lower doses (40 µM), it exerted cytostatic effects, while at higher doses (60 µM), it induced cytotoxicity. Markers of apoptosis and paraptosis were observed, including mitochondrial dilation and ER membrane involvement.	Autophagy and paraptosis induction	[30]
Combinatorial effects of cannabinoid receptor 1 and 2 agonists on characteristics and proteomic alteration in MDA-MB-231 breast cancer cells	2024	Preclinical (in vitro)	MDA-MB-231	Triple-Negative Breast Cancer (TNBC) (ER-, PR-, HER2-)	CB1 and CB2 agonists in a 2:1 ratio (ACEA: GW405833)	Cell proliferation, invasion, lamellipodia formation, proteomic profile alteration	The 2:1 combination prominently inhibited colony formation, induced S-phase cell cycle arrest, and reduced invasion and lamellipodia formation. Proteomic analysis revealed alterations in pathways like ZPR1/SHC1/MAPK and AXL/VAV2/RAC1.	ZPR1/SHC1/MAPK & AXL/VAV2/RAC1 pathways	[31]
Cannabidiol enhances Atezolizumab efficacy by upregulating PD-L1 expression via the cGAS–STING pathway in triple-negative breast cancer cells	2024	In vitro, in vivo	MDA-MB-231	Triple-Negative Breast Cancer (TNBC) (ER-, PR-, HER2-)	Cannabidiol (CBD), various administration methods	PD-L1 expression levels, cGAS-STING pathway activation, tumor cell apoptosis, synergistic effects of CBD and atezolizumab on immune response activation	CBD upregulates PD-L1 expression in TNBC cells via the cGAS-STING pathway, enhancing atezolizumab efficacy. Combination therapy induces stronger anti-tumor immune responses compared to atezolizumab alone	cGAS-STING immune activation, PD-L1 upregulation	[32]
Cannabidiol combination enhances photodynamic therapy effects on MCF-7 breast cancer cells	2024	In vitro (cell lines)	MCF-7	Luminal A Breast Cancer (ER+, PR+, HER2-)	CBD: 1.25, 2.5, 5, 10, and 20 µg/mL (in vitro), Hypericin-Gold nanoparticles, PDT (594 nm, 5 J/cm2)	Cell morphology, LDH release, ATP levels, Trypan Blue exclusion, Immunofluorescence (Cytochrome c, Bcl-2, Bax, p53, PARP)	CBD induced cell death in MCF-7 cells in a dose-dependent manner (vacuolization, blebbing, floating). CBD + PDT combination therapy was effective in killing MCF-7 cells in vitro by induction of apoptosis (increased Cytochrome c, Bax, p53, PARP).	Cytochrome c, Bax, p53, PARP activation	[33]
In vitro evidence of selective pro-apoptotic action of the pure cannabidiol and cannabidiol-rich extract	2023	Preclinical Study	MDA-MB-231 (breast cancer), PC-3 (prostate cancer), MCF-10 A (non-malignant breast cells), PNT2 (non-malignant prostate cells)	Triple-Negative Breast Cancer (TNBC) (ER-, PR-, HER2-) (MDA-MB-231)	0–15 µM pure CBD, CBD-rich Cannabis sativa extracts (extract B and extract D) containing equimolar CBD concentrations	Cell viability, morphological changes, endoplasmic reticulum stress-related apoptosis, gene expression involved in apoptosis and cell cycle control, ROS involvement	Both pure CBD and CBD-rich extracts decreased the viability of MDA-MB-231 and PC-3 cells in a concentration-dependent manner. Endoplasmic reticulum stress-related apoptosis and morphological changes were induced only in low-serum conditions. Non-malignant cell lines (MCF-10 A and PNT2) showed no alterations of viability, suggesting a selective action of CBD in tumor cells. Reactive oxygen species might be involved in the response mechanism. Significant changes in gene expression involved in apoptosis and cell cycle control were observed.	ER stress-related apoptosis, ROS generation	[34]
Rimonabant and Cannabidiol rewrite the interactions between breast cancer cells and tumor microenvironment	2023	In vitro	MCF7, MDA-MB-231	MCF7 (ER positive) and Triple-Negative Breast Cancer (TNBC) (ER-, PR-, HER2-)(MDA-MB-231)	Rimonabant and CBD; specific dosages not mentioned	Tumor-stroma interactions, growth factor secretion, tumor proliferation, angiogenesis, immune reprogramming	Both Rimonabant and CBD altered the release of factors involved in tumor proliferation, angiogenesis, and immune reprogramming, demonstrating anti-metastatic potential by reprogramming the tumor microenvironment	Tumor microenvironment modulation, anti-metastatic potential	[35]
The role of Cannabidiol and tetrahydrocannabivarin to overcome doxorubicin resistance in MDA-MB-231 xenografts in athymic nude mice	2023	In vitro (2D & 3D cultures), In vivo (xenograft model), Transcriptomics, Proteomics	MDA-MB-231	Triple-Negative Breast Cancer (TNBC) (ER-, PR-, HER2-) (MDA-MB-231)	CBD: 2.5–30 µM (in vitro), 10 mg/kg (i.p. in vivo) THCV: 2.5–30 µM (in vitro), 15 mg/kg (i.p. in vivo) Doxorubicin (DOX): 2.5–30 µM (in vitro), 5 mg/kg (i.v. in vivo)	Cell viability (MTT assay in 2D & 3D cultures), Tumor volume (in vivo), RNA sequencing, Proteomics, Western blotting (histone modification markers)	CBD/THCV increased DOX cytotoxicity in DOX-resistant MDA-MB-231 cells (2D & 3D). Downregulated PD-L1, TGF-β, sp1, NLRP3, P38-MAPK, and upregulated AMPK, induced apoptosis. Inhibited H3k4 methylation and H2K5 acetylation.	PD-L1, TGF-β, P38-MAPK inhibition; AMPK upregulation	[36]
Anti-cancer effects of selective cannabinoid agonists in pancreatic and breast cancer cells	2022	In vitro (cell lines)	MDA-MB-231 (TNBC), PANC1 (Pancreatic Cancer)	Triple-Negative Breast Cancer (TNBC) (ER-, PR-, HER2-) (MDA-MB-231)	L-759,633 (CB2 agonist), ACPA (CB1 agonist), ACEA (CB1 agonist): 1-250 µM for 72 h (MTS assay), 50 and 100 µM	Cell proliferation inhibition, clonogenicity suppression, apoptosis induction	CB1/CB2 agonists: Decreased cell proliferation and clonogenicity in both PANC1 and MDA-MB-231 cells. Upregulated pro-apoptotic Bax protein, downregulated anti-apoptotic Bcl-2 protein, induced apoptosis.	Bax upregulation, Bcl-2 downregulation, apoptotic pathways	[37]
Anticancer and chemosensitization effects of cannabidiol in 2D and 3D cultures of TNBC: Involvement of GADD45α, integrin-α5,-β5,-β1, and autophagy	2022	Preclinical (in vitro and ex vivo)	MDA-MB-231, MDA-MB-468, MCF-10 A	Triple-Negative Breast Cancer (TNBC) (ER-, PR-, HER2-)T (MDA-MB-231 and MDA-MB-468), Immortalized Non-Tumorigenic Cells (MCF-10 A)	In vitro: CBD (1-2.5 µM for chemosensitivity, up to 10 µM for cytotoxicity), DOX (0.39 to 25 µM).	Cell viability, cell migration, gene expression (GADD45A, GADD45G, FASN, LOX, Integrins), protein expression (GADD45α, Integrins, Autophagy markers), cell cycle analysis, and organoid dissociation.	CBD showed higher IC50 values in 3D cultures compared to 2D cultures in TNBC cells. CBD alters the expression of GADD45A, GADD45G, FASN, LOX, and integrin genes (α5, β5) in MDA-MB-231 cells. CBD induces anti-migratory effects in TNBC cells by decreasing fibronectin, vimentin, and integrins α5, β5, and β1. CBD inhibits autophagy of TNBC cells by decreasing levels of Beclin1, ATG-5, ATG-7, and ATG-16. CBD pre-treatment increases the sensitivity of TNBC cells to doxorubicin (DOX). CBD induces cell cycle arrest at G0/G1 phase.	GADD45A, Integrins α5/β5 modulation, autophagy inhibition	[38]
Combination of cannabidiol with low-dose naltrexone increases the anticancer action of chemotherapy in vitro and in vivo	2022	Preclinical (in vitro and in vivo)	A549 (human lung cancer), HCT116 (human colorectal cancer), MCF7 (human breast cancer)	ERα+ (MCF-7)	In vitro: CBD (1 µM), LDN (10 nM). NTX (10µM), GEM (~ IC20), OXP (~ IC20); In vivo: LDN (1.2 µg/mouse), CBD (35 µg/mouse), GEM (9 µg/mouse); All in a sequential treatment regimen	Cell number, viability, intracellular signaling protein expression (pAKT, AKT, pERK, ERK, CBR1, CBR2), tumor volume.	LDN before CBD is more effective than CBD before LDN at reducing cell numbers in vitro. LDN/CBD pre-treatment sensitizes cells to chemotherapy. LDN/CBD enhances the effect of gemcitabine in vivo. No significant toxicity was observed in mice treated with LDN/CBD. CBD, LDN and NTX had no significant effect on cell viability as single agents. Both CBR1 and CBR2 are present in all cell lines used. Combination treatments also led to decreased levels of pAKT and pERK in all cell lines.	CBR1/CBR2 modulation, AKT/ERK inhibition	[39]
Activation of cannabinoid receptors in breast cancer cells improves osteoblast viability in cancer-bone interaction model while reducing breast cancer cell survival and migration	2022	Preclinical Study	MDA-MB-231, UMR-106	Triple-Negative Breast Cancer (TNBC) (ER-, PR-, HER2-)T	CB1 and CB2 agonists administered in vitro	Osteoblast viability, breast cancer cell survival, migration	CB receptor activation improved osteoblast viability while reducing breast cancer cell survival and migration; highlighted potential therapeutic benefits in cancer-bone interaction models	CB receptor activation modulates osteoblast-cancer interaction	[40]
Inhibition of cannabinoid receptor type 1 sensitizes triple-negative breast cancer cells to ferroptosis via regulating fatty acid metabolism	2022	Preclinical (in vitro and in vivo)	MDA-MB-231, MDA-MB-436, HCC38, Hs578T, MCF-7, ZR75-1, T47-D, SKBR3, HEK 293 T, HCC1937, BT474, BT549, BT-20, HMEC	Triple-Negative Breast Cancer (TNBC) (ER-, PR-, HER2-), ERα+ (MCF-7)	Rimonabant (CB1 antagonist, concentrations not specified), erastin (ferroptosis inducer, concentrations not specified), RSL3 (ferroptosis inducer, concentrations not specified), LY294002 (PI3K inhibitor, concentrations not specified), PD98059 (MEK inhibitor, concentrations not specified).	Cell viability, lipid peroxide levels, malondialdehyde (MDA) levels, 4-hydroxynonenal (4-HNE) levels, cytosolic ROS production, intracellular glutathione (GSH) depletion, cell cycle analysis, tumor growth (in vivo), RNA sequencing, fatty acid analysis.	Inhibition of CB1 with rimonabant synergizes with erastin/RSL3 to inhibit TNBC cell growth in vitro and in vivo. This synergy is mediated by increased lipid peroxidation, MDA, 4-HNE, and ROS production, and GSH depletion. CB1 inhibition promotes G1 cell cycle arrest. CB1 regulates stearoyl-CoA desaturase 1 (SCD1)- and fatty acyl desaturase 2 (FADS2)-dependent fatty acid metabolism via PI3K-AKT and MAPK signaling pathways, thereby modulating ferroptosis sensitivity in TNBC cells. Dual targeting of CB1 and ferroptosis shows promise as a therapeutic strategy for TNBC.	PI3K-AKT & MAPK signaling, ferroptosis induction	[41]
Improved Therapeutic Efficacy of CBD with Good Tolerance in the Treatment of Breast Cancer through Nanoencapsulation and in Combination with 20(S)-Protopanaxadiol (PPD)	2022	Preclinical (in vitro, in vivo)	4T1	Triple-Negative Breast Cancer (TNBC)	Nanoencapsulated CBD + PPD	Tumor inhibition rate, apoptosis, drug synergy, tolerance profile	Nanoencapsulation improved CBD’s therapeutic effects, achieving 82.2% tumor inhibition, while combination with PPD enhanced anticancer efficacy with good tolerance	Enhanced bioavailability, apoptosis induction	[42]
The Role of Cannabidiol and Tetrahydrocannabivarin to Overcome Doxorubicin Resistance in MDA-MB-231 Xenografts in Athymic Nude Mice	2023	In vitro (2D & 3D cultures), In vivo (xenograft model)	Triple-Negative Breast Cancer (TNBC)	CBD: 2.5–30 µM (in vitro), 10 mg/kg (i.p. in vivo)	Chemosensitivity, apoptosis, immune modulation	CBD/THCV increased DOX cytotoxicity, downregulated immune checkpoint markers such as PD-L1 and TGF-β, overcoming drug resistance	The Role of Cannabidiol and Tetrahydrocannabivarin to Overcome Doxorubicin Resistance in MDA-MB-231 Xenografts in Athymic Nude Mice	PD-L1 & TGF-β downregulation, AMPK activation, histone modification	[36]

**Table 3:** In vivo studies investigating the therapeutic potential of CBD in breast Cancer models

Study Title	Publication Year	Animal Model	Implanted Cells & Site	Carcinogen Use	Mode & Duration of Treatment	Sample Size	Outcomes Measured	Key Findings	References
CBD Inhibits Tumor Development in Breast Cancer Models	2023	Mouse Xenograft Model	MCF-7 cells, subcutaneous implantation	No carcinogen used	Oral administration of CBD at varying doses for 6 weeks	10 mice/group	Tumor growth inhibition, metastasis reduction, apoptosis induction	CBD significantly reduced tumor growth and metastasis	[19]
CBD & THCV Overcome Doxorubicin Resistance in TNBC Xenografts	2023	Athymic Nude Mice Xenograft Model	MDA-MB-231 cells, subcutaneous implantation	No carcinogen used	CBD (10 mg/kg) + THCV (15 mg/kg) + DOX (5 mg/kg) for 6 weeks	10 mice/group	Chemosensitivity enhancement, apoptosis induction, immune modulation	CBD/THCV increased DOX cytotoxicity, downregulated PD-L1 & TGF-β, overcoming resistance	36
CB1 Inhibition Sensitizes TNBC to Ferroptosis via Fatty Acid Metabolism Regulation	2022	Mouse Xenograft Model	MDA-MB-231 cells, subcutaneous implantation	No carcinogen used	Rimonabant (CB1 antagonist) + Erastin/RSL3 for 4 weeks	8 mice/group	Tumor growth reduction, lipid peroxidation, ROS production	CB1 inhibition increased ferroptosis sensitivity and reduced tumor growth	[41]
Enhanced Therapeutic Efficacy of CBD via Nanoencapsulation & Combination with PPD	2022	Mouse Xenograft Model	4T1 cells, subcutaneous implantation	No carcinogen used	Nanoencapsulated CBD + PPD for 5 weeks	12 mice/group	Tumor inhibition, drug synergy, apoptosis induction	Nanoencapsulation improved CBD efficacy (82.2% tumor inhibition) and enhanced anticancer action with PPD	[42]
Anticancer and Chemosensitization Effects of CBD in TNBC Models	2022	Ex Vivo Organotypic Model	MDA-MB-231, MDA-MB-468, MCF-10 A (organoid cultures)	No carcinogen used	CBD (1–10 µM) + DOX (0.39-25 µM) in TNBC 2D/3D models	Not applicable (ex vivo study)	Cell viability, migration, gene expression (GADD45A, Integrins), autophagy markers	CBD improved DOX sensitivity, suppressed TNBC invasion, inhibited autophagy via Beclin1 downregulation	[38]
CBD Enhances Atezolizumab Efficacy via cGAS–STING Pathway Activation in TNBC Models	2024	Mouse Xenograft Model	MDA-MB-231 cells, subcutaneous implantation	No carcinogen used	CBD (dose not specified) + Atezolizumab for treatment duration (not specified)	Sample size not specified	PD-L1 expression, cGAS-STING activation, tumor apoptosis, immune response	CBD upregulated PD-L1 expression via cGAS-STING, enhancing atezolizumab efficacy in TNBC	[32]
Antitumor activity of abnormal cannabidiol and its analog O-1602 in taxol-resistant preclinical models of breast cancer	2019	Zebrafish xenograft model	Paclitaxel-resistant MDA-MB-231 and MCF-7 breast cancer cells	No carcinogen used	Abnormal cannabidiol (Abn-CBD) and O-1602 at varying concentrations (e.g., 2 µM)	Not specified	Cell viability, apoptosis induction, tumor growth inhibition, gene expression analysis	Abn-CBD and O-1602 significantly inhibited proliferation of Taxol-resistant breast cancer cells, induced apoptosis, and reduced tumor growth in xenograft models. Enhanced Taxol’s efficacy via non-CB1/CB2 cannabinoid receptors, suggesting a novel mechanism of action	[29]
Appraising the Entourage Effect: Antitumor Action of a Pure Cannabinoid Versus a Botanical Drug Preparation in Preclinical Models of Breast Cancer	2018	Mouse xenograft model	Breast cancer cell lines implanted subcutaneously	No carcinogen used	Pure cannabinoid and botanical drug preparation administered at varying doses	Not specified	Tumor growth inhibition, apoptosis induction, and gene expression analysis	Botanical drug preparation demonstrated enhanced antitumor activity compared to pure cannabinoid, supporting the entourage effect hypothesis	[28]
Novel Role of Cannabinoid Receptor 2 in Inhibiting EGF/EGFR and IGF-I/IGF-IR Pathways in Breast Cancer	2016	Mouse xenograft model	ERα + and ERα- breast cancer cells implanted subcutaneously	No carcinogen used	JWH-015 (CNR2 agonist) administered at varying doses	Not specified	Tumor growth inhibition, reduced migration and invasion, suppression of EGFR and IGF-IR signaling pathways	CNR2 activation suppressed breast cancer growth through novel mechanisms by inhibiting EGF/EGFR and IGF-I/IGF-IR signaling axes	[27]
Modulation of Breast Cancer Cell Viability by a Cannabinoid Receptor 2 Agonist, JWH-015, is Calcium Dependent	2016	Mouse xenograft model	Murine 4T1 and human MCF-7 breast cancer cells implanted subcutaneously	No carcinogen used	JWH-015 (CNR2 agonist) administered at varying doses	Not specified	Tumor growth inhibition, apoptosis induction, calcium-dependent signaling, MAPK/ERK pathway modulation	JWH-015 significantly reduced tumor burden and metastasis in vivo, induced apoptosis in vitro, and modulated calcium-dependent MAPK/ERK signaling pathways	[26]
Bone Cell-Autonomous Contribution of Type 2 Cannabinoid Receptor to Breast Cancer-Induced Osteolysis	2015	Mouse xenograft model	Bone-tropic human and mouse breast cancer cells implanted in bone	No carcinogen used	CB2 agonists (e.g., HU308, JWH133) administered at varying doses	Not specified	Tumor-induced osteolysis, bone remodeling, osteoblast and osteoclast activity	CB2 activation reduced osteolysis and tumor burden, highlighting its role in regulating tumor-bone interactions and bone remodeling	[25]
Cannabinoids Reduce ErbB2-Driven Breast Cancer Progression Through Akt Inhibition	2010	MMTV-neu mouse model	ErbB2-positive breast cancer cells implanted subcutaneously	No carcinogen used	Δ9-THC and JWH-133 (CB2 agonist) administered at varying doses	Not specified	Tumor growth inhibition, reduced metastasis, apoptosis induction, angiogenesis impairment	Cannabinoids significantly reduced tumor growth, metastasis, and angiogenesis. Their antitumor effects were mediated through Akt pathway inhibition, providing strong preclinical evidence for cannabinoid-based therapies in ErbB2-positive breast cancer	[24]
Synthetic Cannabinoid Receptor Agonists Inhibit Tumor Growth and Metastasis of Breast Cancer	2009	Mouse xenograft model	MDA-MB-231 and MDA-MB-468 breast cancer cells implanted subcutaneously	No carcinogen used	JWH-133 (CB2 agonist) and WIN-55,212-2 (CB1/CB2 agonist) administered at varying doses	Not specified	Tumor growth inhibition, reduced metastasis, apoptosis induction, COX-2/prostaglandin E2 pathway modulation	JWH-133 and WIN-55,212-2 significantly reduced tumor growth (40-50%) and lung metastasis (65-80%) in vivo. Effects were mediated through CB1/CB2 receptors and involved COX-2/prostaglandin E2 signaling pathways	[23]

**Table 4:** Comparison of monotherapy and combination therapy

Aspect	Monotherapy	Combination Therapy
**Efficacy**	Direct cytotoxicity via apoptosis induction (10, 32)	Enhanced chemosensitivity and immune response (9)
**Mechanism**	ROS generation, mitochondrial dysfunction (32)	Synergistic pathway modulation (e.g., TGF-β/PD-L1) (3, 9)
**Clinical Advantage**	Minimal off-target toxicity	Overcomes drug resistance, reduces side effects (42)
**Limitations**	Limited efficacy in advanced tumors (22)	Complexity in dosing optimization (3, 9)

**Table 5:** CBD presents unique advantages compared to traditional breast cancer treatments

Treatment Modality	Advantages	Limitations
**CBD-based Therapy**	Minimal toxicity, potential anti-metastatic effects, modulation of tumor microenvironment	Lack of standardized clinical data, regulatory concerns
**Chemotherapy**	Effective tumor shrinkage, established clinical use	Severe side effects (nausea, neuropathy, immune suppression)
**Targeted Therapy (HER2 inhibitors**,** PARP inhibitors)**	Specific inhibition of cancer-related pathways	High cost, resistance development
**Immunotherapy (PD-1/PD-L1 inhibitors)**	Enhances immune response against tumors	Variable response rates, immune-related adverse events

## Results

### Study selection

The study selection process adhered to the PRISMA 2020 guidelines, as illustrated in Fig. 1. An initial total of 1,191 records were identified through comprehensive searches across multiple databases, including Web of Science (*n* = 112), PubMed (*n* = 59), EMBASE (*n* = 142), Scopus (*n* = 254), and Google Scholar (*n* = 624). After duplicates (*n* = 347), irrelevant automation-marked records (*n* = 187), and records removed for other reasons (*n* = 258) were excluded, 399 records were screened.

### Screening phase

During the screening phase, 206 records were excluded. Of these, 78 were review articles, 14 were conference papers, and 114 were excluded due to lack of relevance to the study’s objectives or focus on cannabidiol (CBD) and breast cancer.

### Eligibility assessment

At the eligibility assessment stage, 193 reports were sought for retrieval, of which 91 were successfully assessed in detail. Among the 102 reports not retrieved, 61 lacked full-text availability, and 41 were removed for other reasons. Ultimately, 91 full-text articles were rigorously evaluated for inclusion criteria.

### Final selection

The final assessment excluded 59 articles for the following reasons:

- 33 studies lacked specific focus on the effects of CBD on breast cancer.
- 24 studies either analyzed other cannabinoids or did not include desired indices relevant to the research aims.

Ultimately, 34 studies met the rigorous inclusion criteria and were incorporated into this systematic review. The final selection process ensured that only high-quality, relevant studies contributed to the analysis, providing a robust overview of the therapeutic potential of cannabidiol (CBD) in breast cancer.

## Discussion

### Therapeutic potential of Cannabidiol (CBD) in breast Cancer

Cannabidiol (CBD) has emerged as a promising therapeutic agent for breast cancer, exhibiting diverse anticancer properties, including apoptosis induction, inhibition of proliferation, suppression of metastasis, and modulation of the tumor microenvironment [1, 43]. Preclinical studies highlight the effectiveness of CBD in reducing tumor burden and limiting metastatic potential, particularly in triple-negative breast cancer (TNBC)—a highly aggressive subtype with limited targeted therapies [2, 10].

Below, we synthesize key findings to evaluate CBD’s therapeutic potential, molecular mechanisms, and clinical implications, supported by evidence from preclinical and clinical studies.

### CBD as monotherapy in breast Cancer

CBD has demonstrated antitumor properties across multiple breast cancer subtypes, including ER + and TNBC. Key mechanisms and findings include:

### Mechanisms of action

- **ROS Generation and ER Stress**: CBD induces apoptosis via reactive oxygen species (ROS) generation and endoplasmic reticulum stress, disrupting mitochondrial dynamics and redox balance in cancer cells [32].
- **Pathway Modulation**: CBD inhibits PI3K/AKT and MAPK signaling pathways, suppressing cell proliferation and metastasis. For instance, CBD downregulates Id-1, a key regulator of tumor aggressiveness, in MDA-MB-231 cells [10].
- **Selective Toxicity**: CBD exhibits selective cytotoxicity toward cancer cells while sparing normal cells. For example, CBD-loaded microparticles showed extended antiproliferative activity in MCF-7 and MDA-MB-231 cells without harming non-cancerous counterparts [32].

### Mitochondrial dysfunction

CBD modulates mitochondrial dynamics, inducing metabolic stress and apoptosis. In MCF-7 cells, CBD disrupted mitochondrial redox balance, leading to caspase-3 activation and DNA fragmentation [32, 42].

### CBD in combination therapy

CBD synergizes with conventional therapies to enhance efficacy and overcome drug resistance:

- **Doxorubicin Resistance**: Co-administration of CBD with doxorubicin (DOX) reduced TGF-β and PD-L1 expression, reversing chemoresistance in TNBC models [9].
- **Taxol Resistance**: Abnormal cannabidiol (Abn-CBD) enhanced Taxol’s efficacy in Taxol-resistant MDA-MB-231 cells by inducing apoptosis via non-CB1/CB2 pathways [29].

Table 4 outlines the key distinctions between CBD monotherapy and its combination with other anticancer agents. As presented in the table, combination therapy has demonstrated enhanced chemosensitization and immune response modulation, whereas monotherapy primarily exerts its effects through apoptosis induction.

### Immunomodulation

- **PD-L1 Upregulation**: CBD enhances PD-L1 expression via the cGAS-STING pathway, improving the efficacy of immune checkpoint inhibitors like Atezolizumab in TNBC [3, 32].

### Photodynamic therapy (PDT)

Combining CBD with PDT significantly increased apoptosis in MCF-7 cells through oxidative stress pathways, suggesting a dual mechanism of action [42].

### Drug delivery systems for CBD in breast Cancer therapy

In preclinical studies evaluating CBD as a monotherapy, various drug delivery systems have been employed to enhance its bioavailability and efficacy:

#### Oral administration

- **Bioavailability Challenge**: CBD has low oral bioavailability due to extensive first-pass metabolism.
- **Potential Solutions**: Nanoencapsulation and lipid-based formulations enhance absorption.

#### Intravenous (IV) delivery

- **Advantages**: Allows precise dosing, bypasses metabolism, and achieves rapid systemic circulation.
- **Limitations**: Requires specialized formulations such as liposomal CBD or CBD nanoparticles.

#### Nanoencapsulation & nanoparticle systems

- **Improved Therapeutic Efficacy**: Studies have demonstrated that nanoencapsulated CBD significantly increases tumor inhibition rates (e.g., 82.2% in murine models).
- **Combination Potential**: Used alongside PPD, nanoparticles improve CBD’s anticancer effects and tolerance.

#### Transdermal & topical applications

- **Localized Targeting**: Could be useful in reducing inflammation and tumor-associated pain.
- **Limitations**: Poor penetration into deeper breast tissues.

#### Inhalation routes

- **Rapid Absorption**: Inhaled CBD quickly enters systemic circulation, potentially bypassing metabolic barriers.
- **Concerns**: Variability in dosing and absorption rates.

#### Combination with other drug delivery technologies

- **Photodynamic Therapy (PDT)**: Studies show CBD enhances PDT-induced apoptosis in breast cancer cells via oxidative stress pathways.
- **Chemotherapy Adjuncts**: CBD combined with Doxorubicin, Atezolizumab, and Taxol enhances drug efficacy while mitigating resistance.

#### Direct administration

- Some in vitro studies involve the direct addition of CBD to cell cultures, providing precise control over drug concentration.

### Clinical implications and future directions

While preclinical data are compelling, clinical translation requires addressing critical gaps:

1. **Dosing Optimization**: Standardized protocols for CBD administration (e.g., micellar formulations for targeted delivery).
2. **Subtype-Specific Effects**: Further research on CBD’s role in hormone receptor-positive breast cancer, particularly its interaction with ERα and AR signaling.
3. **Metastasis Prevention**: Investigate CBD’s impact on cancer stem cells and metastatic pathways (e.g., ZPR1/SHC1/MAPK and AXL/VAV2/RAC1).
4. **Safety Profiling**: Large-scale trials to assess long-term safety, drug interactions, and effects on non-cancerous tissues.

### Challenges and controversies

Despite promising evidence, several challenges hinder CBD’s clinical application in breast cancer treatment:

• **Variability in Study Design**: One of the primary concerns surrounding CBD research is the inconsistency in dosing regimens, administration routes, and experimental models used across studies. For instance, McAllister et al. (2007) demonstrated that CBD inhibits tumor progression through Id-1 gene suppression, but variations in drug concentration and cell line-specific responses complicate direct comparisons [8].
• **Regulatory and Legal Barriers**: The classification of cannabinoids under regulatory frameworks affects the pace of clinical investigations. While THC-containing formulations face tighter restrictions, CBD’s legal status varies globally, impeding its widespread clinical adoption [7].
• **Pharmacokinetics and Drug Interactions**: CBD’s metabolic pathways involve cytochrome P450 enzymes, which can lead to potential drug interactions when combined with conventional chemotherapeutics [5]. Understanding its pharmacokinetics is crucial for optimizing therapeutic applications.

### Research gaps and future directions

There are notable gaps in the current literature that need to be addressed to facilitate the clinical transition of CBD in breast cancer treatment:

• **Limited Clinical Trials**: While preclinical models provide strong evidence for CBD’s anticancer activity, clinical trials evaluating its efficacy and safety in breast cancer patients remain scarce [6]. Large-scale randomized controlled trials are necessary to validate preclinical findings.
• **Biomarker Identification**: Predictive biomarkers for CBD responsiveness need to be identified to facilitate personalized cancer therapy. This includes assessing potential correlations with cannabinoid receptor expression (CB1, CB2) and genetic alterations in tumor cells [32].
• Long-Term Safety and Toxicity: Comprehensive studies investigating CBD’s long-term effects on tumor recurrence, immune responses, and systemic toxicity are essential for its clinical integration [28].

### Comparative analysis of CBD-Based therapies and conventional treatments

Table 5 provides a comparative analysis of CBD-based therapy against conventional breast cancer treatments, including chemotherapy, targeted therapy, and immunotherapy. As the table illustrates, CBD’s potential advantages—such as its minimal toxicity and modulation of the tumor microenvironment—make it a promising therapeutic candidate, despite the current challenges related to standardization and regulatory frameworks.

CBD’s potential to enhance sensitivity to chemotherapy and immunotherapy represents an area of growing interest. For example, Kalvala et al. (2023) demonstrated that CBD can overcome doxorubicin resistance in TNBC models by downregulating immune checkpoint pathways such as PD-L1 [36]. Additionally, CBD’s ability to modulate inflammatory cytokines suggests its use as an adjunct to standard therapies in preventing therapy-induced toxicity [16].

## Conclusion

CBD holds significant promise as a complementary or standalone therapeutic agent in breast cancer treatment, particularly in TNBC, where conventional options are limited. However, clinical validation through well-designed trials, biomarker identification, and safety profiling remains imperative before widespread clinical adoption. Future studies should focus on optimizing combinatorial therapies, investigating long-term effects, and refining pharmacological formulations to bridge the gap between preclinical findings and clinical application.

By addressing these challenges, CBD could potentially redefine breast cancer management strategies, offering a safer, more effective, and targeted approach to treatment.

## Data Availability

No datasets were generated or analysed during the current study.

## Abbreviations

AEA: Anandamide (N-arachidonoylethanolamine)
AKT: Protein Kinase B
AMPK: AMP-activated protein kinase
AR: Androgen Receptor
ATG: Autophagy-related Gene
BCRP: Breast Cancer Resistance Protein
CB1/CB2: Cannabinoid Receptors 1 and 2
CBD: Cannabidiol
CNR2: Cannabinoid Receptor 2
COX-2: Cyclooxygenase-2
DOX: Doxorubicin
EGFR: Epidermal Growth Factor Receptor
ER: Estrogen Receptor
ERK: Extracellular signal-Regulated Kinase
FASN: Fatty Acid Synthase
GADD45: Growth Arrest and DNA Damage-inducible protein
HER2: Human Epidermal Growth Factor Receptor 2
IGF-IR: Insulin-like Growth Factor I Receptor
JWH-015: Cannabinoid Receptor 2 Agonist
MAPK: Mitogen-Activated Protein Kinase
MMP: Matrix Metalloproteinase
mTOR: Mechanistic Target of Rapamycin
PDT: Photodynamic Therapy
PD-L1: Programmed Death-Ligand 1
PI3K: Phosphoinositide 3-Kinase
PPARγ: Peroxisome Proliferator-Activated Receptor Gamma
ROS: Reactive Oxygen Species
SCID: Severe Combined Immunodeficiency (mice model)
SMA: Styrene Maleic Acid
STAT3: Signal Transducer and Activator of Transcription 3
TNBC: Triple-Negative Breast Cancer
TRPV1: Transient Receptor Potential Vanilloid 1
VEGF: Vascular Endothelial Growth Factor
